# Diagnostic concordance of remote WSI-based thyroid cytology compared to conventional cytological diagnosis: a pilot study

**DOI:** 10.1186/s13000-026-01820-9

**Published:** 2026-09-22

**Authors:** Abdullah Fahri Şahi̇n, Özben Yalçin, Seyma Büyücek, Merve Dogan Ayan, Hatice Elmas

**Affiliations:** 1 Department of Pathology, School of Medicine, Malatya Turgut Ozal University, Malatya, Türkiye; 2 University of Health Sciences Turkey, Istanbul Prof. Dr. Cemil Tascioglu City Hospital, Istanbul, Türkiye; 3 Section Cytopathology, Institute of Pathology, University Medical Center Hamburg-Eppendorf UKE, Hamburg, D-20246 Germany; 4 Airway Research Center North (ARCN), German Center for Lung Research (DZL), Giessen, Germany

**Keywords:** Thyroid fine-needle aspiration, Bethesda system, Whole slide imaging, Digital pathology, Telepathology

## Abstract

**Background:**

Whole slide imaging (WSI) has emerged as a key component of digital pathology, enabling remote diagnostics and standardized evaluation. However, its diagnostic concordance in thyroid cytopathology, particularly under real-world conditions, remains insufficiently validated.

**Methods:**

In this retrospective pilot study, thirteen thyroid cases were evaluated using both conventional light microscopy and WSI. One pathologist performed glass slide evaluation, while two independent pathologists assessed the corresponding digital slides remotely. All evaluations were conducted in a blinded manner. Diagnostic agreement was analyzed using percentage concordance and Cohen’s kappa coefficient.

**Results:**

The overall concordance rate between conventional microscopy and WSI was 69.2%. Cohen’s kappa values ranged from 0.58 to 0.63, indicating moderate to substantial agreement. Full concordance was observed in 7 cases, predominantly among malignant diagnoses. Partial concordance occurred in 5 cases, mainly involving borderline categories. Complete discordance was identified in 1 case, exclusively within an indeterminate cytological category. No clinically significant misclassification between benign and malignant diagnoses was observed.

**Conclusion:**

WSI demonstrated moderate diagnostic agreement with conventional microscopy in well-defined thyroid cytopathology categories. However, variability remains more pronounced in indeterminate cases, reflecting inherent diagnostic challenges. These findings support the preliminary feasibility of WSI in real-world and remote diagnostic workflows, although larger, multicenter studies are required to confirm these results.

## Introduction

The global epidemiology of thyroid disease has undergone a profound transformation over recent decades, marked by a substantial increase in the detection of thyroid nodules. This apparent rise is largely attributable to the widespread use of high-resolution ultrasonography and the consequent identification of incidentalomas rather than a true increase in disease prevalence [1, 2]. As a result, contemporary thyroid oncology faces the critical challenge of distinguishing indolent lesions from clinically significant malignancies in order to minimize both overtreatment and missed diagnoses.

Fine-needle aspiration biopsy (FNAB) remains the cornerstone of preoperative risk stratification in thyroid nodules, offering a minimally invasive, cost-effective, and widely accessible diagnostic approach [3]. However, despite its well-established clinical utility, FNAB is inherently limited by sampling variability, operator dependence, and interpretative subjectivity [4, 5]. The implementation of The Bethesda System for Reporting Thyroid Cytopathology has significantly improved diagnostic standardization; nevertheless, it has not eliminated the intrinsic ambiguity associated with indeterminate diagnostic categories [6, 7].

In particular, the “Atypia of Undetermined Significance” (AUS/FLUS) and “Suspicious for Malignancy” categories continue to represent diagnostic grey zones, characterized by considerable interobserver variability and heterogeneous malignancy risk [5, 8]. These uncertainties frequently translate into clinical management dilemmas, potentially leading to either unnecessary surgical interventions or inadequate follow-up strategies.

In this context, digital pathology—and specifically whole slide imaging (WSI)—has emerged as a transformative technological paradigm with the potential to redefine cytopathology workflows. By enabling high-resolution digitization and remote accessibility of cytological specimens, WSI facilitates standardized interpretation and enhances reproducibility across institutions [9, 10]. Furthermore, WSI serves as a key enabler of telepathology, allowing subspecialty consultation and second-opinion workflows that may significantly influence clinical decision-making [10].

Moreover, the integration of digital pathology with artificial intelligence (AI) and advanced image analysis approaches has demonstrated substantial potential in improving diagnostic concordance, reducing interobserver variability, and enabling more objective interpretation in cytopathology workflows [11, 12].

Despite these advantages, the implementation of WSI in cytopathology presents unique technical challenges compared to histopathology. Cytological preparations exhibit a complex three-dimensional architecture, including overlapping cellular clusters and variable thickness, requiring multi-focal (Z-stacking) acquisition for optimal visualization [9]. These technical requirements significantly increase scanning time, data storage demands, and computational burden, thereby limiting large-scale adoption in routine practice [10, 13].

In addition, the performance of WSI in cytopathology may be influenced by the type of cytological preparation and staining protocol used. Direct smears, liquid-based cytology (LBC), and cell block preparations present distinct morphological and technical characteristics that may affect digital image acquisition and interpretation. Similarly, staining methods such as Papanicolaou and May–Grünwald–Giemsa provide different cytomorphological details and may influence observer performance in the digital environment. In the present study, routinely prepared H&E-, Papanicolaou-, and Giemsa-stained slides were evaluated according to case availability, reflecting real-world diagnostic practice.

Although multiple studies have demonstrated high diagnostic concordance between WSI and conventional light microscopy, the available evidence remains heterogeneous and is predominantly derived from high-resource academic centers [10]. Consequently, the College of American Pathologists (CAP) continues to emphasize the necessity of rigorous validation studies prior to routine clinical implementation, including standardized protocols with adequate case numbers and defined washout periods [13].

Notably, there remains a significant gap in the literature regarding the implementation and validation of WSI in thyroid cytopathology within emerging healthcare systems. In middle-income countries, digital pathology adoption is often constrained by infrastructural, economic, and regulatory limitations, as well as the lack of locally generated validation data (9). This gap restricts the integration of digital workflows into routine clinical practice and limits the scalability of telepathology networks.

Against this background, the present study was designed to address this critical gap by evaluating the diagnostic concordance between conventional microscopy and WSI in thyroid cytopathology within a real-world clinical setting. By employing a structured validation approach aligned with CAP recommendations, this study aims to provide preliminary evidence regarding the feasibility of digital pathology in routine diagnostic workflows.

## Materials and methods

### Study design and setting

This retrospective observational concordance study was conducted at the Pathology Laboratory of Malatya Turgut Özal University Training and Research Hospital. In addition, thyroid cases originating from the University of Health Sciences Turkey, Istanbul Prof. Dr. Cemil Taşcıoğlu City Hospital were included in the study.

The primary aim was to evaluate the diagnostic concordance between conventional light microscopy and WSI in thyroid pathology within a real-world clinical workflow.

### Case selection and specimen characteristics

A total of 13 thyroid cases were retrospectively selected for this study. All cases included in the primary analysis consisted of cytological specimens obtained via FNA, in line with the study’s objective of evaluating diagnostic concordance in thyroid cytopathology. Cases were selected to represent a spectrum of routine cytopathological diagnostic categories encountered in clinical practice. Histopathological confirmation was not available for all cases, and therefore was not used as a uniform reference standard in the primary analysis.

Cases were retrospectively selected from routine thyroid FNA specimens evaluated between June 2025 and February 2026. The study cohort was intentionally assembled to include a spectrum of diagnostic categories encountered in routine practice, including benign, indeterminate, and malignant cases. Cases with insufficient material, unavailable digital images, or incomplete clinical data were excluded. Specimen adequacy was assessed according to routine cytopathological practice and Bethesda reporting criteria.

The conventional cytological diagnosis established by glass-slide evaluation was used as the primary reference standard. WSI interpretations were compared against this reference to assess diagnostic concordance.

Histopathological outcomes were available for a subset of cases and were used for secondary descriptive analysis to provide preliminary insight into diagnostic alignment.

### Specimen transfer and handling

For the purpose of digitalization, glass slides prepared at Malatya Turgut Özal University were securely transported via medical courier to Prof. Dr. Cemil Taşcıoğlu City Hospital in Istanbul, covering a distance of approximately 1,100 km.

After completion of the scanning process, all specimens were returned to the originating institution under controlled conditions. This bidirectional transfer was conducted in accordance with institutional protocols to ensure specimen integrity, traceability, and data security throughout the study.

This workflow reflects real-world logistical conditions encountered in institutions without on-site WSI infrastructure and highlights the feasibility of implementing remote digital pathology solutions in resource-limited settings.

### Digital slide acquisition

All glass slides were digitized using a Hamamatsu NanoZoomer S360 whole-slide scanner (Hamamatsu Photonics, Hamamatsu, Japan) at ×20 magnification. Z-stacking was not used. Scanning parameters were standardized by the manufacturer and adapted to the routine workflow of the participating laboratory, generating high-resolution digital images suitable for diagnostic evaluation.

Digital slides were accessed remotely via AnyDesk remote desktop software and reviewed using the Hamamatsu digital pathology viewing platform.

Approximately 4–6 representative H&E-, Papanicolaou-, and/or Giemsa-stained slides were evaluated per case.

### Pathologist evaluation protocol

Three board-certified pathologists participated in the study:

Pathologist 1 performed the reference evaluation using conventional light microscopy (glass slides) on-site.

Pathologists 2 and 3 independently evaluated the same cases using WSI in a remote setting.

All evaluations were conducted independently and in a blinded manner, with no access to prior diagnoses or to each other’s interpretations.

During case review, the participating pathologists had access to the biopsy identification number, patient age, sex, radiological findings, and thyroid function test results (T3, T4, and TSH), reflecting the information routinely available in clinical practice. However, they had no access to the reference diagnosis or to each other’s interpretations.

The primary analysis of this study was based on cytological evaluations, with the aim of comparing diagnostic interpretations obtained via conventional light microscopy and WSI using the same cytological preparations.

### Remote access and data security

Digital slides were accessed remotely via AnyDesk remote desktop software. To ensure patient confidentiality and compliance with data protection regulations, each access session was individually authorized and supervised by a designated technical staff member at Prof. Dr. Cemil Taşcıoğlu City Hospital.

This controlled access framework ensured secure and standardized use of the digital pathology system.

### Digital platform and viewing environment

All digital slide evaluations were performed using the Hamamatsu (NanoZoomer S360) digital pathology viewing platform, which allows high-resolution image visualization, navigation, and magnification comparable to conventional microscopy.

### Statistical analysis

Statistical analyses were performed to assess diagnostic agreement between observers and modalities:

- Cohen’s kappa coefficient (κ) was used to evaluate interobserver agreement.
- Percentage concordance (%) was calculated to determine overall diagnostic consistency.

Kappa values were interpreted according to standard criteria:

< 0.20 (poor), 0.21–0.40 (fair), 0.41–0.60 (moderate), 0.61–0.80 (substantial), and > 0.80 (almost perfect agreement).

## Results

A total of 13 thyroid cases were included in the study, demonstrating a marked female predominance (92.3%, *n* = 12) with a mean age of 47.5 years (range: 30–60 years). Table 1 summarizes the radiological findings of the included cases together with the corresponding conventional microscopy diagnoses, WSI interpretations by two independent observers, and concordance patterns. Most nodules exhibited heterogeneous and/or multinodular morphology, frequently accompanied by cystic components. Hypoechogenicity was the most common ultrasonographic feature, while microcalcifications were observed in a subset of cases.

**Table 1:** Radiological findings and diagnostic comparison between conventional microscopy and WSI in thyroid cytology cases (*n* = 13)

Case	Radiological Findings	Reference (Glass Slide)	Observer 1	Observer 2	Agreement Pattern
1	Hypoechoic nodules measuring 34 × 26 mm in the right lobe and 41 × 27 mm in the isthmus	Suspicious for a follicular neoplasm	Benign	Suspicious for a follicular neoplasm	Partial
2	Multiple nodular structures throughout the thyroid gland, largest measuring 7 mm	Benign	Benign	Benign	Full
3	Well-defined nodules: 11 × 11 mm in the right lobe and 12 × 11 mm in the left lobe	Suspicious for Malignancy	Malignant	Malignant	Partial
4	Calcified cystic heterogeneous nodule measuring 29 × 14 mm in the right lobe	PTC microcarcinoma	PTC microcarcinoma	PTC microcarcinoma	Full
5	Heterogeneous hypoechoic nodule with microcalcifications, 14 × 13 mm (right lobe)	Papillary Thyroid Carcinoma	Papillary Thyroid Carcinoma	Papillary Thyroid Carcinoma	Full
6	Cystic degenerative nodule with macrocalcifications, 26 × 22 mm (right lobe)	Follicular nodular hyperplasia + lymphocytic thyroiditis	Same	Same	Full
7	Multiple isoechoic nodules; largest 15 mm (right) and 16 mm (left)	Malignant cytology (PTC)	Same	Same	Full
8	Heterogeneous nodule with cystic and solid components, largest 7 mm	Benign cytology	Benign	Non-diagnostic	Partial
9	Multinodular thyroid with cystic areas; nodules measuring 34 × 26 mm (right lobe) and 41 × 27 mm (isthmus)	AUS	Follicular neoplasm	Benign	Discordant
10	Heterogeneous hypoechoic nodules with cystic areas, largest 19 × 28 mm (left lobe)	Benign cytology with Hürthle cell change	Benign	AUS	Partial
11	Hypoechoic solid nodule with microcalcifications, 9 × 7 mm (right lobe–isthmus junction)	Suspicious for Malignancy	AUS	Suspicious for Malignancy	Partial
12	Hypoechoic solid nodule with microcalcifications, 9 × 7 mm (right lobe–isthmus junction)	PTC microcarcinoma	Same	Same	Full
13	Multiple heterogeneous hypoechoic nodules with small cystic areas in the left lobe (15 × 11 mm, 4 × 3 mm) and calcified cystic hypoechoic nodule (4 × 7 mm) in the right lobe	Papillary Thyroid Carcinoma	Same	Same	Full

**Fig. 1:**
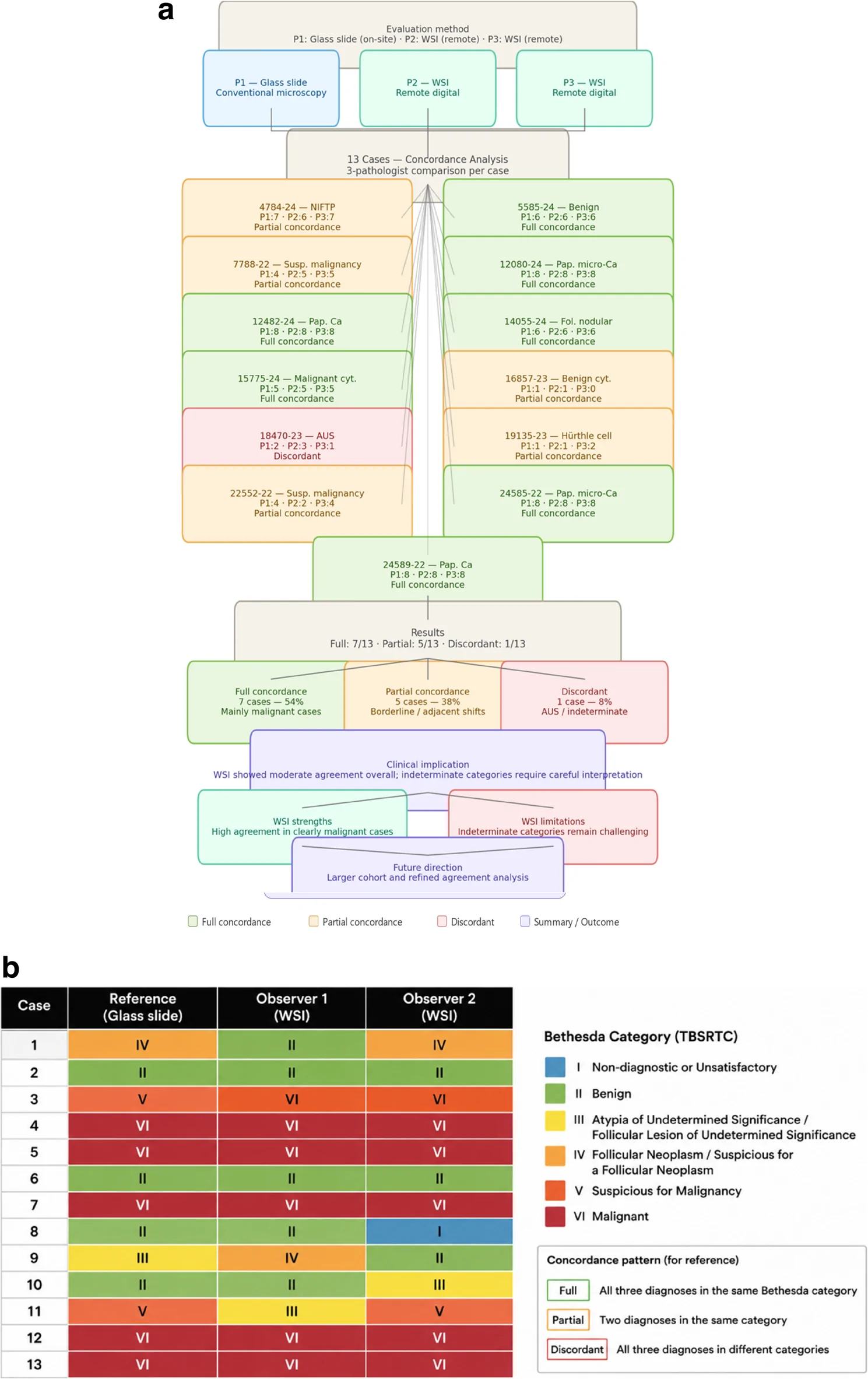
Diagnostic workflow and interobserver concordance analysis. Figure 1 illustrates the evaluation workflow and agreement patterns among three pathologists (P) assessing thyroid cytology cases using conventional microscopy and whole slide imaging (WSI). Cases are categorized as full concordance, partial concordance, or discordant. Full agreement was predominantly observed in malignant cases, whereas discrepancies were mainly confined to indeterminate categories such as AUS and suspicious for malignancy. Case-wise distribution of Bethesda System for Reporting Thyroid Cytopathology (TBSRTC) categories across conventional microscopy and whole-slide imaging (WSI) observers. Heatmap illustrating the Bethesda category assigned to each of the 13 thyroid cytology cases by conventional microscopy (reference glass-slide diagnosis) and by two independent observers using whole-slide imaging (WSI). Colors correspond to Bethesda categories I–VI and enable visual comparison of diagnostic classifications across observers. Concordant cases are characterized by identical category assignments, whereas partial and discordant cases demonstrate shifts between adjacent or distinct Bethesda categories. Most disagreements were observed within indeterminate categories, particularly atypia of undetermined significance (AUS/FLUS), follicular neoplasm, and suspicious for malignancy, while malignant categories showed the highest level of agreement.

The overall diagnostic workflow and interobserver agreement patterns are illustrated in (Fig. 1). Final diagnoses comprised a spectrum of entities, including malignant lesions (*n* = 6), predominantly papillary thyroid carcinoma, benign lesions (*n* = 3), a low-risk tumor classified as Suspicious for a follicular neoplasm (Bethesda IV) (*n* = 1), and indeterminate cytological categories (*n* = 3). The reference diagnostic categories were based on conventional cytological evaluation.

Diagnostic agreement between conventional light microscopy and WSI was assessed across all cases. Observer 1 and Observer 2 each demonstrated concordance with the reference diagnosis in 9 of 13 cases (69.2%). When stratified by diagnostic category, concordance was highest in malignant cases and remained high in benign cases, whereas most discrepancies occurred within indeterminate categories, including AUS, follicular neoplasm, and suspicious for malignancy. Cohen’s kappa values were approximately 0.63 (95% CI: 0.33–0.93) for Pathologist 1 and 0.58 (95% CI: 0.28–0.88) for Pathologist 2, indicating moderate to substantial agreement.

As demonstrated in Figure 1, three distinct patterns of agreement were identified across the study cohort. Full concordance was observed in 7 cases, predominantly corresponding to malignant diagnoses, all showing complete agreement among observers. Partial concordance was identified in 5 cases, mainly involving borderline or intermediate categories, where minor shifts between adjacent diagnostic groups were noted. Complete discordance was observed in 1 case within an indeterminate cytological categor

Discrepancies were primarily characterized by shifts between adjacent diagnostic categories, such as benign to nondiagnostic, AUS to follicular neoplasm, or suspicious to malignant. No direct discordance between clearly benign and malignant categories was observed in this limited cohort. No clinically significant misclassification between benign and malignant categories was observed.

These findings suggest that WSI demonstrated moderate agreement with conventional microscopy in clearly defined categories, while variability appeared to be more pronounced in indeterminate cytological interpretations.

## Discussion

The present study evaluated the diagnostic concordance between conventional light microscopy and WSI in thyroid cytopathology within a real-world clinical setting. The findings demonstrated a moderate to substantial level of agreement between the two modalities, with an overall concordance rate of 69.2% and kappa values ranging between 0.58 and 0.63. These results are broadly consistent with previously published studies reporting high concordance between WSI and conventional microscopy in cytopathology [14].

A key observation of this study is that diagnostic agreement was highest in clearly defined categories, particularly malignant and benign cases [2, 14]. This finding aligns with existing literature, which suggests that WSI performs reliably in cases with well-established morphological features [1]. In contrast, discrepancies were predominantly observed in indeterminate cytological categories, including AUS/FLUS and suspicious for malignancy. This pattern is well-recognized in thyroid cytopathology and reflects the intrinsic interpretative variability associated with these categories rather than limitations specific to the digital modality [4, 7].

Importantly, no direct discordance between clearly benign and malignant categories was observed in this cohort. Although the limited sample size precludes definitive conclusions, this finding provides preliminary support for the feasibility of WSI in routine diagnostic workflows, particularly for clinically decisive categories. Similar observations have been reported in previous validation studies, indicating that digital pathology could be considered for primary diagnosis under controlled conditions [15]. In this context, WSI not only enables remote evaluation but also represents a critical infrastructure for the future implementation of AI-driven decision support systems in cytopathology, and has been shown to be feasible and clinically applicable in telepathology-based consultation systems [16, 18]. In particular, this capability allows cases to be reviewed by subspecialty experts across different institutions and may be especially beneficial for peripheral or resource-limited centers where access to experienced cytopathologists is limited.

The observed discrepancies were primarily characterized by shifts between adjacent diagnostic categories. This finding underscores the well-documented challenges associated with borderline cytological interpretations, where even conventional microscopy demonstrates significant interobserver variability [5, 19]. Therefore, the variability observed in this study should be interpreted within the broader context of cytopathology rather than attributed solely to WSI.

From a technical perspective, the use of WSI in cytology remains more challenging than in histopathology due to the three-dimensional architecture of cytological specimens and the need for adequate focus across multiple planes. Limitations related to single-plane scanning and the absence of Z-stacking may contribute to diagnostic uncertainty in certain cases. These technical constraints have been previously highlighted as barriers to widespread adoption of digital cytopathology [9, 13].

Despite these challenges, WSI offers several practical advantages for cytopathology, including remote access to cases, telepathology consultation, digital archiving, and support for multidisciplinary case discussions. Furthermore, the integration of artificial intelligence and advanced image analysis tools has the potential to improve diagnostic consistency and workflow efficiency. Nevertheless, implementation barriers remain, including high infrastructure costs, large data storage requirements, increased scanning times, and technical limitations related to the evaluation of three-dimensional cytological specimens. These factors should be considered when planning the broader adoption of digital cytopathology in routine practice [2, 10, 13].

These technical and logistical factors may partly explain the variability observed in indeterminate cytological categories in the present study, particularly in AUS and suspicious for malignancy groups. In our study setting, several real-world constraints may have further contributed to this variability. First, the absence of an in-house WSI scanner at the primary institution necessitated the physical transfer of glass slides to an external center for digitization. This process introduced an inherent delay, with transportation requiring approximately one day. Although the study included a total of 13 cases, a cumulative total of 45 slides (including H&E, Papanicolaou, and/or Giemsa-stained preparations) were transferred and evaluated, reflecting the complexity of cytopathological assessment. Additionally, due to the large file size of digital slides—typically ranging between 2 and 3 GB per slide—and the presence of multiple representative slides per case (approximately 4–6 slides per patient), direct data transfer posed significant technical and logistical challenges. Therefore, remote access via secure desktop-sharing platforms (e.g., AnyDesk, Zoom) was utilized as a practical, cost-effective, and reliable alternative to dedicated commercial WSI viewing systems. This approach represents a pragmatic and resource-adapted telepathology model, reflecting real-world implementation scenarios in settings where dedicated WSI infrastructure is not readily available [10]. While this strategy enabled successful remote diagnostics, such constraints may have influenced workflow efficiency and interpretative conditions, thereby contributing to variability in borderline cytological categories. However, this workflow should be considered a transitional and context-specific solution rather than a sustainable routine model, underscoring the need for local WSI infrastructure to enable scalable implementation. Recent experiences from countries with established national digital cytopathology programs further demonstrate how standardized quality assurance systems, WSI integration, and digital education platforms can facilitate large-scale implementation of digital cytopathology workflows [20, 21]. Recent advances in AI, machine learning, and radiomics have further expanded the potential of digital pathology, enabling more objective and reproducible diagnostic workflows. AI-assisted image analysis has been shown to enhance classification accuracy, reduce interobserver variability, and facilitate integration of morphological, radiological, and molecular data in oncological diagnostics [11]. In this context, WSI not only enables remote evaluation but also represents a critical infrastructure for the future implementation of AI-driven decision support systems in cytopathology, and has been shown to be feasible and clinically applicable in telepathology-based consultation systems [16–18].

Previous studies have also demonstrated the feasibility and diagnostic reliability of rapid remote evaluation systems in cytopathology, supporting the use of digital platforms for real-time or near-real-time diagnostic workflows [22].

The use of conventional microscopy as the reference standard may introduce bias, as it reflects routine clinical diagnosis rather than an independent gold standard. This study has several limitations that should be acknowledged. First, the sample size is relatively small, and the findings should therefore be considered preliminary. Second, the study design reflects a pilot validation approach and may not fully capture the variability encountered in larger, more diverse cohorts. Third, although histopathological outcomes were available for some cases, the primary analysis was based on cytological interpretation, which may limit direct comparison with outcome-based diagnostic assessment. The relatively wide confidence intervals observed for the kappa estimates reflect the limited sample size and indicate substantial statistical uncertainty. Although the point estimates suggested moderate to substantial agreement, the confidence intervals spanned a broad range of agreement levels. Therefore, the findings should be interpreted with caution and require confirmation in larger validation studies.

These findings provide preliminary evidence supporting the technical feasibility of remote WSI-based assessment in thyroid cytopathology [23–27]. The inclusion of remote evaluation and multi-observer assessment further strengthens the practical relevance of the findings, particularly in the context of telepathology and resource-limited settings.

In conclusion, this pilot study demonstrated the technical feasibility of WSI-based evaluation and moderate agreement with conventional microscopy in thyroid cytopathology. Variability remained more pronounced in indeterminate cases. Further large-scale, multicenter studies are warranted to validate these findings and to better define the role of digital pathology in routine cytological practice.

## Conclusion

This pilot study suggests that WSI demonstrated a moderate level of agreement with conventional light microscopy in thyroid cytopathology, particularly in clearly defined benign and malignant categories.

Diagnostic variability was primarily observed in indeterminate cytological categories, such as atypia of undetermined significance and suspicious for malignancy, reflecting the inherent subjectivity of cytopathological interpretation rather than limitations specific to the digital modality.

Although no direct discordance between benign and malignant diagnoses was identified in this limited cohort, the small sample size necessitates cautious interpretation of these findings.

Overall, WSI appears to be a feasible tool in thyroid cytopathology within a pilot, real-world setting. These findings highlight the potential of WSI to bridge gaps in access to subspecialty expertise, particularly in resource-limited settings. Further large-scale, multicenter studies are required to validate these preliminary findings and to establish standardized protocols for routine clinical implementation.

## Data Availability

No datasets were generated or analysed during the current study.
